# Social support utilization’s effect on post-traumatic stress symptoms: a Danish cross-sectional study of 383 ambulance personnel

**DOI:** 10.3389/fpsyt.2024.1425254

**Published:** 2024-07-31

**Authors:** Pernille Melander, Maria Louison Vang, Nanna Lindekilde, Lars Peter Sønderbo Andersen, Ask Elklit, Jesper Pihl-Thingvad

**Affiliations:** ^1^ Department of Clinical Research, University of Southern Denmark, Odense, Denmark; ^2^ Department of Occupational and Environmental Medicine, Odense University Hospital, Odense, Denmark; ^3^ National Centre for Psychotraumatology, Department of Psychology, University of Southern Denmark, Odense, Denmark; ^4^ Danish Ramazzini Centre, Department of Occupational Medicine – University Research Clinic, Goedstrup Hospital, Herning, Denmark

**Keywords:** social support, PTSS, PTSD, ambulance personnel, work environment, help seeking, trauma, first responders

## Abstract

**Introduction:**

Social support is considered an important factor in prevention of mental illness. However, little is known about the association between ambulance personnel’s use of multiple types of social support and post-traumatic stress symptoms (PTSS). This study aims to assess if number of used social support types predicts PTSS for ambulance personnel. Apart from assessing the main effect of social support utilization, we were interested in investigating if social support utilization moderated the effect of frequency of critical events on PTSS.

**Materials and methods:**

A total of 383 ambulance personnel completed a survey consisting of validated questionnaires. Hierarchical multiple linear regression analyses were performed to assess the association between frequency of traumatic exposure and utilization of social support and PTSS as outcome variable.

**Results:**

Higher number of utilized social support types was associated with higher levels of PTSS (β = 0.15, p <.001). When serving as a moderator of the association between frequency of exposure to critical incidents and PTSS, social support utilization had a significant and positive interaction effect (β = 0.26, p = .049). 307 participants had used 2 or 3 types of informal support during the past year, whereas 81 had used 2 or 3 types of formal support.

**Conclusion:**

To our knowledge, this is the first study investigating the relationship between utilization of multiple, concurrent social support types and PTSS. This study suggests that to understand the effects of social support among ambulance personnel, it is necessary to assess the utilization of multiple concurrent support types, contributing factors to social support use, and different patterns of social support utilization that constitutes professional life in ambulance work.

## Introduction

Ambulance work is marked by routine exposure to traumatic events such as life-threatening situations and experiences of human suffering (1–3), and has thus been marked as a high-risk profession due to frequent exposure to critical incidents (4). Critical incidents in the context of ambulance work can be defined as distressing events that overwhelm or threaten to overwhelm an individual’s coping strategies (5). Ambulance personnel respond to a higher number of emergency calls compared to other first responders, increasing their potential distress (6, 7). The high level of exposure to critical incidents due to the nature of their operational work, puts this professional group at an increased risk of developing post-traumatic stress disorder (PTSD) (1, 2, 8). PTSD is defined by symptoms such as re-experiencing the event, avoidance of traumatic memories and a heightened sense of threat at a level that compromises the individual’s functioning (9). The estimated prevalence of PTSD among ambulance personnel has been estimated to be approximately 11% (10), compared to 4% in the general population (11), indicating an elevated risk of PTSD among ambulance personnel. Investigating and preventing the development of PTSD in this group thus seem highly relevant.

The everyday operational duty of ambulance work has been found to be as stressful as disaster work in emergency professions (7). This can appear in the shape of less sensational incidents, such as lonely deaths or emotional involvement with patients, which may pose an increased risk of overwhelming emotional responses (3). It is theorized that frequent exposure to less severe trauma over time is associated with an elevated risk of developing PTSD (12, 13). This has been elaborated in studies finding that frequency of exposure to operational work of lower severity, aggravating circumstances, and less severe tragic events are potential risk factors for PTSD in ambulance work (3, 14, 15). The need to attend to the effects of being exposed to incidents of lower arousal over longer time courses, and not only the effects of high arousal situations such as mass fatalities has previously been suggested for this professional group (15, 16). Therefore, it seems crucial to consider both high arousal situations and everyday operational events when examining the risk of psycho-traumatic stress responses.

The association between to critical incidents and the development of PTSD is a significant concern for personnel. It is important to note that ambulance personnel, like other first responders, are a specialized and highly selected group, expected to react differently to critical incidents compared to civilians (17). The risk of delayed onset PTSD and fluctuating symptomatology is higher among professionals, potentially masked by habituation and training (18–20). Ambulance personnel’s habituation of exposure to critical incidents has been suggested to lead to a progressive risk of PTSD over time (21). Further, expression of avoidance and hyper-arousal symptoms can be more subtle due to habituation and training (22). Consequently, despite their perceived hardiness, ambulance personnel demonstrate high scores of PTSS when compared to the general population and other first responders (8, 23, 24). As a result, professionals might experience PTSS long before the fulminate expression of clinical PTSD. Moreover, they are at risk of being continuously exposed to critical incidents even while they experience initial stages of PTSD development. This highlights the necessity to examine the risk and protective factors specific to ambulance personnel in the development of PTSD. By considering these multifaceted aspects of exposure and their impact on mental health, a more comprehensive understanding of the development of PTSS among ambulance personnel can be gained.

That ambulance personnel are at risk of developing PTSS is apparent, although detecting PTSS development can be complicated by habituation and a long and fluctuating prodromal phase. It is therefore important to investigating factors relevant for the associations between exposure to critical incidents and PTSS prior to the full onset of clinical PTSD.

Containing trauma-related symptoms has previous been regarded as a prerequisite in ambulance work (25, 26). It has been suggested that professionals learn to contain their emotional response to stressors in order to function at work due to job strain and continuous exposure (27). For individuals exposed to trauma, Ehlers & Clark presented that actively trying not to think about the traumatic event was a common maladaptive cognitive strategy (28). The action differs from thought suppression by using cognitive preoccupation with other things instead of pushing thoughts of the trauma away. Both strategies are regarded as detrimental to the integration of the traumatic incident and thus recovery (28). Specifically for ambulance personnel, a tendency to distract oneself from the memories of the incident in order to maintain a professional distance, can contribute to maintaining PTSS (29).

Social support is recognized as a significant factor in preventing PTSD and promoting resilience in trauma-exposed individuals (30–32).

It has been suggested that lack of social support generally increase the risk of PTSD among professionals (2). Nevertheless, reluctance towards seeking support have been highlighted as a tendency among ambulance personnel (5, 26, 33, 34). This reluctance has been attributed to fear of stigma by peers and managers (5, 34), fear of being deemed weak by the work place (5, 26, 33), fear of having one’s confidentiality broken by team managers (26, 33), fear of burdening peers (33), as well as lack of time at work to seek support and not knowing where to get proper help (35). Apart from enhancing emotional and cognitive processing through social support (36), the purpose of utilizing support can also be to gain instrumental support. Yet, qualitative studies have indicated that it is unlikely for emergency service personnel to request instrumental support themselves, thus not gaining a needed downtime period, alternative work schedules, or avoiding being called to specific accidents (25). This could possibly maintain an increased job strain and vulnerability towards developing PTSS. Qualitative studies have highlighted that ambulance personnel often prefer to confide in close confidants at work and rely on informal support networks for empathetic reactions (37–39). Informal support has also been found to be a preferred style of support rather than formalized peer support among ambulance personnel. Fear of being judged or sanctioned by the workplace is highlighted as a reason not to engage in formal support (25, 40). Therefore, ambulance personnel can be expected to use the most accessible sources of support, e.g. *ad hoc* support via established, informal relations, rather than formal support types.

Prospective studies of first responders, including ambulance personnel show no or small effect of social support, when measured as perceived social support (41, 42) and social function (43) following single, catastrophic events (41, 43) One of the few longitudinal studies of ambulance personnel found positive correlations between lack of support from colleagues and managers and mental health symptoms, although effects on PTSS were weak and mostly insignificant (44). This study did not, however, measure support use, but assessed perceived lack of support at work. A cross-sectional study of ambulance personnel investigating the effects of perceived and received social support found that only perceived social support showed significant effects on PTSS, concluding that perceived social support is a stronger predictor of PTSS than received support (45). None of the measures had a moderating effect on the association between organizational stressors, including emotionally taxing or stressful minor incidents, and PTSS (45). Received social support was measured with the Inventory of Socially Supportive Behaviors–Short Form (ISSB) (46). The ISSB offers an estimation of the number of supportive actions received by the respondent over a period of time, but does not offer insights into who and how many the respondent has received support from. Research on social support among first responders, including ambulance personnel, have primarily focused on perceived social support and general attitudes towards support, following catastrophic events, as well as combining social support with other comping mechanisms, necessitating further investigation into the effects of utilizing multiple types of social support on PTSS specifically among ambulance personnel. Several of the existing studies on social support among ambulance personnel have included multiple types of emergency work, such as firefighting, police work, and ambulance work (34, 47), not focusing specifically on the risk and conditions of the ambulance profession.

To assess the specific risk of PTSD for ambulance personnel, studies are needed that focus solely on specific conditions for this professional group. Understanding the development of PTSS in ambulance work requires considering everyday exposure to operational work and the concurrent use of various support types. To our knowledge, no studies have been made measuring the effects of using multiple support sources of social support on PTSS specifically for ambulance personnel. This could be relevant in order to clarify if the personnel have sought support and if this is associated with decreased PTSS symptomatology. The objective of this study is thus to investigate if use of number of social support types is associated with PTSS among ambulance personnel. Additionally, it investigates whether social support utilization moderates the effect of critical incident frequency on PTSS. By examining the associations between social support utilization and PTSS, we propose that valuable insights can be gained into the potential role of support in mitigating the impact of critical incidents on the mental health of ambulance personnel.

The hypotheses proposed for this study are as follows:

1) A higher level of social support is associated with lower levels of PTSS,

2) The association between high exposure to critical incidents and PTSS is moderated by the utilization of social support,

3) Informal support types are more commonly used than formal support types among ambulance personnel.

## Materials and methods

### Participants and procedures

This cross sectional survey study is part of the prospective cohort study *You Don’t Stand Alone* (14), investigating critical incidents and development of PTSD in a Danish ambulance organization. The current study is based on the baseline sample. All operational ambulance personnel (N = 703) in a large public ambulance organization were invited to the survey. The organization represents 21.5 percent of all 3271 Danish ambulance personnel, including ambulance rescuer students (48). The sample consists of ambulance personnel in operative duty with functions as emergency service transport personnel, ambulance rescuer students, ambulance rescuer assistants, ambulance rescuers, and paramedics. Managers were not included as operational ambulance personnel, as their engagement in operational duty is lower than the employees and as their utilization of support was expected to be different than the employees.

The respondents are employed at a station in one of the seven areas in the organization, apart from a minority employed across stations. In total, 703 ambulance personnel were invited and 453 chose to participate. 408 completed the baseline survey, rendering a response rate of 58%. Further, 25 respondents had missing data in either the main outcome or explanatory variable, rendering a sample of 383 respondents (54.5% of the study population).

### Ethics

Participants were informed of the purpose and nature of the survey though an online information sheet and participation was based on written consent. The project complies with GDPR requirements (the Danish Data Protection Authority, # 20/47381). The study was presented to the Scientific Ethics Committee, receiving the formal response that according to Danish law, the study was not subject to approval by the committee (# 20222000–78).

### Measurements

#### Outcome variable

##### PTSS

PTSS was measured using the validated Danish version of the International Trauma Questionnaire (ITQ), 6-item version, rated on a five-point Likert scale from “Not at all” (0) to “Extremely” (4) (49, 50). The scale measures PTSS during the past month with two items for each of the three symptom clusters of PTSD: re-experiencing, avoidance, and hypervigilance (49).

The scale has shown both good construct validity (51) and criterion validity in different trauma populations (52). Compared to the *DSM-5*, it has been found to produce significantly lower diagnostic rates (53), which is considered relevant to reduce the risk of over-reporting in a non-clinical population. ITQ has been recommended specifically for assessing PTSS among ambulance personnel due to the construct and phrasing of symptoms that resonates with the population’s work exposure (54). The overall symptom level of PTSS was assessed by summing the six items into a sum scale from 0–24. PTSD among first responders is expected to develop over long time courses and with fluctuating symptomatology due to ongoing exposure (18–20). We therefore chose to measure PTSS over clinical cases of PTSD to assess the prodromal symptoms of a working population. The scale showed good internal consistency with Cronbach’s Alpha = 0.79.

#### Explanatory variables

##### Social support

To assess the effects of social support utilization on PTSS, we were interested in measuring how many different types of social support the ambulance personnel had engaged in the aftermath of critical incidents at work the past year, specifically targeted at the population of ambulance personnel and the support types available to our population. To target the population and the support types available to them, we used a modified version of the General Help Seeking Questionnaire (55), asking about the use of specific sources of formal and informal support applicable to the ambulance context. The respondents were asked to report which types of support they had used following critical incidents during the past year.

In total 10 support types, both within the organizational and private context, were measured. Three questions covered types of informal support (informal collegial support, informal managerial support, and support from spouse or close friends) and seven questions covered types of formal support (debriefing/defusing, formal peer support by a colleague trained in providing support, formal support by a manager trained in providing support, crisis psychologist through work, psychologist outside of work, general practitioner, other health professionals). Answers were given as yes/no for each type of support. All items were then summed to generate a variable disclosing the total use of types of support.

For *post hoc* analyses, we were interested in investigating how different levels of utilized support was associated with PTSS. We therefore defined variables based on sample distribution and number of questions on the scale to ensure meaningful grouping of the respondents. This rendered subcategories of low (0 -2 types of support), medium (3–4 types), and high support utilization (5 or more types of support).

To assess potentially different effects of how the support was accessed, we divided the scale into two variables of support utilization: a variable based on the informal support types and a variable based on formal support types. Each variable of informal and formal support were leveled into categories, also based on the sample distribution and number of support types on the subscales, resulting in number of support types of the low, medium and high categories different from the variable of total support utilization. The informal support group thus consisted of three categories, low support (0–1 type), medium (2 types) and high (3 types). The formal support utilization was coded into three categories, no formal support (0 types), low (1 type), and medium or high formal support utilization (2 or more types).

#### Measure of exposure

##### CISAW-D

To assess the exposure to critical incidents at work, we used a validated questionnaire developed for ambulance personnel: Critical incidents scale for ambulance work – Denmark (14). The 28-item questionnaire represents the diversity of critical incidents, ambulance personnel in Denmark are exposed to, covering themes from child suicide, to errors made by one self, or a colleague causing damage to the patient (14).

For each type of traumatic event, the respondents were asked to assess how many times they have been exposed to the stressor the past year, reported on an interval scale from the value 0 (zero times) to 5 (more than 20 times the past year). To assess the cumulative exposure to all critical incidents the past year, the scale was summed providing a sum scale from 0–140.

#### Possible confounders

We included a single item on the individual’s appraisal of intensity of this year’s critical incidents at work to account for the overwhelming character of the event and its potential effect on PTSS. The item on intensity of the most critical event at work was answered using an adapted version of the regret intensity scale (56). The respondents were asked to evaluate the most emotionally taxing critical event at work the past year and to assess how strong an emotional impression the event had on them, answering on a scale from 1 = no emotional impression to 10 = very strong emotional impression.

Based on existing literature on risk factors for PTSD, we included confounding factors related to demographics and life style. We included age and gender as a potential confounder in our preliminary analysis as a safety measure, as the factors have been highlighted as a potential risk factor of PTSD in general (57). Seniority has been found a potential risk factor in previous ambulance studies (15, 58), so we included a measure of years in service.

Also, a measure of alcohol consumption was included to account for potential abuse, as this has been positively correlated with PTSD in a comparable sample (59). Alcohol was measured with the validated item from the Danish Psychosocial Questionnaire (60). Here the respondents were asked to assess the daily intake of sum of alcohol units on average, measured on a six item-scale from 0 to 5 or more alcohol units per day.

Potential trauma exposure outside of work, both within the past year and throughout life was chosen as potential confounders, because earlier traumatization is a predictor for PTSD (61). Trauma exposure outside of work was assessed with the validated scale from the national comorbidity study (62). The scale consists of 15-items, 14 items on typical critical incidents that can cause trauma reactions, and one item on “other critical or overwhelming incidents”. The scale was summed and treated as a scale ranging from 0–15. For assessment of potential trauma exposure the past year, the same scale and procedure was carried out, but without the items on trauma exposure in childhood and framed to only include incidents occurring the past year, thus resulting in a scale from 0–13.

To adjust for possible effects of trying to manage demands in daily life with continuous exposure to critical incidents at work, we included the item “Have you tried just to forget about the incident?” The question differs from avoidance symptoms in the sense that thought avoidance as a PTS symptom is associated with individual distress (63). In our survey, the question on trying to forget about the event could be answered although the respondent found the event less or not stressful. Also, the question could be answered regardless of the PTSS. The item was answered by a yes/no response, where the respondent was prompted to answer based on their experience with the past year’s most distressing event. The single item was used in *post hoc* analysis to assess the effect of a possible distancing coping strategy.

#### Statistical methods

Visual representation of the data’s normal distribution, linearity and homoscedasticity by distribution graphs, QQ-plots and residual plots were used to assess data assumptions for linear modelling. Linear correlation of the variables was assessed with Pearson’s r-estimation and variance inflation factor. Descriptive analyses were performed of the single variables in order to estimate means, standard deviations and range or distribution in categories (N and percentages).

Preliminarily, bivariate analyses were performed with each potential confounder and PTSS as outcome, and in multiple regression analyses with social support utilization as an additional explanatory variable and PTSS as outcome. Confounders were selected based on their effect on the model measured by statistical significance and change in adjusted R squared (64). The criterion for adjusted R squared was set at < 10% to prevent over adjustment of the model (65).

To test our hypotheses 1, 2 and 4, we performed stepwise linear regression with the primary explanatory variable, number of used social support types, at step one and PTSS as outcome. In step two, a multiple linear regression model was performed, including the selected, confounding factors. To test our second hypothesis, step three of the model included an interaction effect frequency of critical incidents * number of used social support types with PTSS as outcome, adjusted for all confounders.

To assess the effects of the specific form of utilized support and investigate potential findings from our moderation analysis, we performed two multiple regression analyses with summed informal and formal support as numerical variables, respectively, as *post-hoc* procedures. We then assessed the effects of the different levels of social support use, based on stratified analysis, where we used category variables (low, medium and high) across all support types, as well as for use of informal support types (low, medium and high) formal support types (no, low and medium/high) respectively with the group of lowest support usage set as referent. Levene’s test indicated that the assumption of homogeneity of variance had been violated for groups of total number of total support utilization, (F (2, 380) = 4.47, p <.011), number of informal support types (F (2, 380) = 4.25, p <.01), as well as formal support types, (F (2, 380) = 5.45, p <.005). The analyses were therefore performed with bootstrapped one-way ANOVA. Games-Howell analyses were performed to assess differences between group means.

As a robust measure, the analyses were performed both with pairwise deletion (N=408) and list-wise deletion (N=383) of cases with missing values in explanatory or outcome variables. As our analyses are performed using subscales grouping the respondents into smaller groups, we chose to base our analyses on the completed cases only to secure strength of data.

All analyses were done with R-studio, version 2023.06.

## Results

### Assessment of data

Visual representation of our data showed that the outcome of PTSS was right-skewed. This violation of the model’s basic assumption was accounted for by using bootstrapping with 1000 resamples (66).

Three outliers were observed and investigated with subset analyses without the outliers. Outliers were assessed to be plausible answers and were kept in the main analysis. Sensitivity analysis indicated that exclusion of the outliers had no substantial effect on the results.

Prior to the main analyses, simple regression analyses investigating potential confounders were conducted. Age, gender, seniority, alcohol consumption, trauma outside of work during the past year and throughout life did not contribute sufficiently to the models, and were therefore excluded from the main analyses.

Correlation tests with Pearson’s R revealed low-moderate correlations for all variables chosen for main analyses. All correlations were significant in the range very weak to moderate (.11-.45) except from the correlations between “trying to forget about the incident” and informal support and intensity of this year’s most critical incident respectively (see Appendix A). Variance inflation factor values ranged from 1.02 – 1.24, indicating low risk of multicollinearity (66).

### Descriptive results

Most of the respondents were either trained as paramedics (31.1%), ambulance rescuer (38.4%) or ambulance rescuer assistant (13.3%), and represented the characteristics of the reference population, although with slightly less responses from the emergency service transport personnel.

The overall distribution of respondents across areas corresponded with the reference population overall, except from two areas in which there were an underrepresentation and one area with overrepresentation (see **Table 1**).

**Table 1:** Presenting descriptive data of the sample and reference population in percentages (N) and mean (SD).

	Respondents (N = 383)	Population (N = 703)
Gender Male Female	82% (n=314) 18% (n=69)	577 (82.1) 126 (17.9)
Area Area 1 Area 2 Area 3 Area 4 Area 5 Area 6 Area 7 Other	11.7% (n = 45) 8.9% (n = 34) 16.7% (n = 64) 24% (n = 92) 12% (n= 46) 11.2% (n = 43) 12% (n = 46) 3.4% (n = 13)	16.8% (n = 118) 10.0% (n = 70) 19.8% (n = 139) 19.8% (n = 139) 10.8% (n = 76) 12.7% (n = 89) 10.2% (n = 72) -
Work function EMS transport personnel Ambulance rescuer student Ambulance rescuer assistant Ambulance rescuer Paramedic	8.1% (n = 31) 9.1% (n = 35) 13.3% (n= 51) 38.4% (n= 147) 31.1% (n= 119)	11.4% (n = 80) 9.25% (n = 65) 11.9% (n = 85) 37.4% (n = 263) 29.9% (n = 210)
Age	42.01 (9.75)	42.46 (10.70)
Seniority (years)	14.08 (9.84)	––––
Posttraumatic stress symptoms (PTSS)	3.04 (3.27)	––––
Frequency of critical incidents	28.89 (16.44)	––––
Intensity of this year’s most traumatizing event at work	5.86 (2.41)	––––

The average respondent was male with 14.08 years of experience in the emergency services (SD=9.85) with 82% male and 18% female respondents. The mean age was 42.01 years (SD = 9.72). The sample corresponded to the employee population in terms of gender and age (see **Table 1**). The average score of PTSS across the sample was relatively low (M=3.04, SD = 3.27, range = 0–21), which was expected both due to the zero inflation of the outcome and the fact that our sample was drawn from a working population. A total of 23.2% reported no symptoms of PTSS. All the respondents had been exposed to a critical event the past year (see **Table 1**).

### Main results

#### Hypothesis 1: High level of utilized social support is associated with higher levels of PTSS

Step 1 of the main analysis showed that usage of a higher number of support types was significantly, and positively associated with higher levels of PTSS (see **Table 2**). Thus, use of more support types was associated with higher level of PTSS, albeit with a relative low mean PTSS score. The association was consistent when adjusted for confounders in step 2, although the effect attenuated. The model significantly explained 36% of the variance of PTSS.

**Table 2:** Hierarchical regression analysis of support utilization and confounders on the dependent variable PTSS (N=383).

	Model 1					Model 2					Model 3				
Variable	B	SE B	β	t-statistics	p	B	SE B	β	t-statistics	p	B	SE B	β	t-statistics	p
Intercept	0.86	0.30	- *	2.52	.012	-2.15	0.42	- ***	-5.35	<.001	-1.30	0.58	- *	-2.23	.026
Number of utilized social support types	0.77	0.11	0.35***	7.24	<.001	0.34	0.10	0.15***	3.42	<.001	-0.00	0.20	-0.00	-0.00	.999
Intensity of this work year’s most traumatizing event						0.42	0.06	0.31***	6.83	<.001	0.43	0.06	0.32***	6.94	<.001
Frequency of critical incidents						0.04	0.01	0.22***	5.03	<.001	0.01	0.02	0.06	0.62	.536
Actively trying not to think of this year’s trauma at work						2.06	0.36	0.27***	6.48	<.001	2.09	0.32	0.27***	6.60	<.001
Frequency of critical incidents x Number of support sources utilized											0.01	0.01	0.26*	1.97	.049
R²		.12					.36					.37			

*values significant at p<.05, *** values significant at p<.001.

#### Hypothesis 2: The association between high exposure to critical incidents at ambulance rescue work and PTSS is moderated by number of utilized social support

The moderation analyses in model 3 (see **Table 2**) showed social support utilization significantly moderated the association between frequency of exposure’s association and PTSS with a positive direction of the moderation effect. This model explained 37% of the variance of PTSS.

#### Hypothesis 3: Informal support types are more used than formal support types among ambulance personnel

Summaries of how many had utilized each type of support revealed that the informal support types overall were more utilized over formal types (see **Table 3**). Informal collegial support was the most utilized type of support with 349 respondents having utilized this following a critical incident during the past year. The use of informal managerial support was reported by 135 respondents. In comparison, 62 reported use of formal managerial support and use of formal collegial support was reported by 40 respondents.

**Table 3:** Presenting distribution of utilized support measured by the single items of Help Seeking Questionnaire (N=383).

Type of social support source	N (%)
Informal managerial support • Yes • No	• 134 (35) • 249 (65)
Formal managerial support • Yes • No	• 62 (16.2) • 321 (83.8)
Informal collegial support • Yes • No	• 349 (91.1) • 34 (8.88)
Formal collegial support • Yes • No	• 40 (10.4) • 343 (89.6)
Debriefing or defusing • Yes • No	• 99 (25.8) • 284 (74.2)
Crisis psychologist provided by the work place • Yes • No	• 23 (6) • 360 (94)
Psychologist accessed outside of the work place • Yes • No	• 35 (9.1) • 348 (90.9)
General practitioner • Yes • No	• 23 (6) • 360 (94)
Family or close friends • Yes • No	• 289 (75.5) • 94 (24.5)
Other types of treatment (psychotherapist, physical therapist, alternative treatment, etc.) • Yes • No	• 39 (10.2) • 344 (89.8)

A majority of the respondents had used more than two types of support with 164 having used three or four different types, and 50 having used five or more types of support. A total of 307 had used more than one type of informal support, whereas only 82 had utilized more than one type of formal support. A total of 188 had not used any types of formal support (see Appendix B).

#### *Post-hoc* analysis – stratifying support utilization

To investigate further patterns behind our findings from hypothesis one and two, we performed stratified analyses of social support use. This in order to assess if the effect were driven by the utilization of a certain level of support use, or by informal or formal support utilization. **Table 3** shows the results from the multiple regression analyses with informal and formal support utilization instead of total support utilization.

In the multiple regression analyses, formal support utilization had a small and statistical significant effect on PTSS. Informal support utilization as primary explanatory variable showed no significant association with PTSS (see **Table 4**).

**Table 4:** Hierarchical regression analysis for variables predicting PTS with formal support in model 4 and 5 (N=383) and number of informal support types in model 6 and 7 (N=383).

Variable	Model 4					Model 5				
	B	SE B	β	t-statistics	p	B	SE B	β	t-statistics	p
Intercept	2.23	0.19	–	10.99	<.001	-1.75	0.41	–	-4.60	<.001
Number of utilized formal social support types	0.97	0.17	0.32***	6.49	<.001	0.05	0.15	0.16***	3.70	<.001
Intensity of this work year’s most traumatizing event						0.45	0.06	0.33***	7.46	<.001
Frequency of critical incidents						0.04	0.01	0.23***	5.19	<.001
Actively trying not to think of this year’s trauma at work						1.98	0.36	0.26***	6.19	<.001
R²	.10					.37			
Variable	Model 6					Model 7				
	B	SE B	β	t-statistics	p	B	SE B	β	t-statistics	p
Intercept	1.23	0.44	–	2.83	.004	-1.98	0.44	–	-4.45	<.001
Number of utilized informal social support types	0.90	0.20	0.22***	4.48	<.001	0.22	0.18	0.06	1.21	.228
Intensity of this work year’s most traumatizing incident						0.47	0.06	0.34***	7.44	<.001
Frequency of critical incidents						0.05	0.01	0.23***	5.23	<.001
Actively trying not to think of this year’s trauma at work						2.17	0.32	0.29***	6.76	<.001
R²	.05					.35				

Values significant at p ≤ 0.001***.

In order to compare the effects of levels of the total number of utilized support, as well as number of utilized formal support and informal support, we chose to perform analyses of both subtypes of support. The *post hoc* analysis of total support utilization showed a clear exposure response pattern between the different groups of support utilization and level of PTSS with the groups of higher utilization showing higher levels of PTSS (see **Table 5**). The level of PTSS was statistically different between low, medium, and high level of total support utilization.

**Table 5:** Estimated means and mean differences of PTSS between low, medium or high social support utilization (low, medium or high), N=383.

	Estimated means	SE	95 CI	p
Low number of total support utilization	2.09***	0.57	[1.6 – 2.6]	<.001
Medium number of total support utilization	3.38***	0.58	[2.7 – 4.1]	<.001
High number of total support utilization	5.12 ***	0.53	[4.1 – 6.1]	<.001
	Contrast estimates	SE	95 CI	p
Low vs. Medium number of total support utilization	1.29***	0.23	[0.5 – 2.1]	<.001
Low vs. High number of total support utilization	3.03***	0.40	[1.6 – 4.4]	<.001
Medium vs. High number of total support utilization	1.74*	0.43	[0.3 – 3.2]	.016

T-statistics of estimates are significant at p ≤ 0.05^*^, 0.001^***.^

Results indicated a similar exposure response pattern across the three groups of formal support utilization with an increasing mean level of PTSS in the groups with higher utilization of formal support (see **Table 6**). Comparing the groups showed highly statistically significant differences between the no support group and the low and medium/high group, respectively. The estimated mean difference of PTSS were statistical significant different between the no and low support group and low vs. medium/high formal support utilization group.

**Table 6:** Estimated means and mean differences of PTSS between no, low or medium/high formal social support utilization, N=383.

	Estimated means	SE	95 CI	p
No number of formal support	2.25***	0.18	[1.8 – 2.7]	<.001
Low number of formal support	3.25**	0.38	[2.5 – 4.0]	<.001
Medium or high number of formal support	4.56***	0.45	[3.7 – 5.4]	<.001
	Contrast estimates	SE	95 CI	p
No vs. Low number of formal support	1.00*	0.27	[0.1 – 1.9]	.029
No vs. Medium or high number of formal support	2.31***	0.31	[1.3 – 3.4]	<.001
Low vs. Medium or high number of formal support	1.31*	0.37	[0.1 – 2.6]	.039

T-statistics of estimates are significant at p ≤ 0.05^*^, 0.01^**^, 0.001^***^.

The results from the three groups of informal support utilization showed exposure response pattern similar to the formal utilization variable (see **Table 7**). Comparing the groups, however, only showed highly statistically significant differences between the low support group and the medium and high group respectively. The estimated mean difference of PTSS was not statistical significantly different between the groups of medium and high informal support utilization.

**Table 7:** Estimated means and mean differences of PTSS (contrast estimates) between low, medium or high informal social support utilization (low, medium or high), N=383.

	Estimated means	SE	95 CI	p
Low number of informal support utilization	1.76***	0.28	[1.0 – 2.5]	<.001
Medium number of informal support utilization	3.09**	0.36	[2.2 – 3.9]	<.001
High number of informal support utilization	3.85***	0.45	[2.9 – 4.8]	<.001
	Contrast estimates	SE	95 CI	p
Low vs. medium number of informal support utilization	1.33***	0.25	[0.5 – 2.2]	<.001
Low vs. high number of informal support utilization	2.09***	0.33	[0.9 – 3.2]	<.001
Medium vs. high number of informal support utilization	0.75	0.30	[-0.3 – 1.8]	.189

T-statistics of estimates are significant at p ≤ 0.01**, 0.001***.

## Discussion

Our main objective was to assess whether utilization of support types was a relevant factor for PTSS levels for ambulance personnel. Apart from the main effect of social support utilization, we were interested in assessing if the use of social support moderated the effect of frequency of critical events on PTSS. Unlike previous studies, we used a measure of how many different types of social support the respondent had used following critical incidents the past year, both informal and formal source at work and in their personal life. This was chosen to investigate what types of social support, ambulance workers use and if it is attained through formal channels or via *ad hoc*, informal interaction. Further, we wanted insights into which types of support were more used, and if frequency of critical incidents in ambulance work was associated with level of PTSS.

We found a positive association between utilization of support and PTSS, indicating increased levels of PTSS as more support types are used. Similarly we found that social support utilization moderates the effect of frequency of critical events on PTSS in a positive direction. Informal support types were more used than formal support. Frequency of critical incidents in ambulance work was associated with level of PTSS.

### Social support utilization is associated with higher levels of PTSS

For our first hypothesis, we found that increased utilization of support types was positively associated with higher levels of PTSS, when adjusting for other risk factors in ambulance work. A similar pattern has been found in a Danish cohort of civilians (67). Results from existing studies are ambiguous on the association between social support and PTSS in first responders and overall contrasts our findings.

Low social support has been associated with higher level of PTSS for paramedic trainees (59) and firefighters (68).

Past studies of social support and PTSS in first responder cohorts including ambulance personnel found no association between social support and PTSS both following single critical incidents (41) and as a result of the daily work (41, 42). Here, social support was measured as perceived social support (41, 42).

We suggest that these contrasts from our results are partially due to the fact that we measured number of utilized support types, thus reflecting the symptomatology associated with how many different types of support sources, one has engaged following a critical incident. The results might thus indicate that the more symptoms of PTSS are present, the higher incentive to seek support.

Other studies have measured social support through social function scales, thus providing the respondents’ assessment of function, regardless of actual used support (7, 43) or accessibility of support sources (69). In van der Ploeg & Kleber’s study (44), only social support at work was assessed through questions reflecting perceived social support. They found that social factors were main risk factors of mental health outcomes, and that lack of support from the supervisor was positively related to PTSS, however lack of collegial support was not. Apart from one study (70) studies in this area have not accounted for different levels of social support use. It is however unclear what exact measure is used in Maslow et al. (70) and whether they assess both private and professional support types.

The Crisis Support Scale (71) applied in two of the studies (41, 42) assesses the perceived support and accessibility of support following a critical incident, but does not specify which or how many sources that have been engaged. The Social Adjustment Scale (72) assess function more broadly, e.g. whether one maintains contact with friends as usual, maintains work function, etc., and not specifically if the respondent has talked about the critical incident or sought social support due to the critical incident. Measures of the perceived quality of social support would probably have an inverse effect on PTSS as studies have shown that the experience of lack of support is a probable etiological factor for PTSD (31).

Further, there is a possibility that the reversed findings may be due to relations between exposure, symptom development and social support utilization which could be established through a longitudinal design.

The contrasting finding may therefore reflect the highlighting of different aspects of social support and PTSS. To our knowledge, our choice of measuring support utilization, rather than perceived support or intentions to seek help, highlights a dynamic between use of support and PTSS among ambulance personnel that until now has been under-examined in this area of research. We argue that the results and the contrast to existing literature indicates a need for awareness of the multitudes of social support types that constitutes the daily relations of a professional in combination with the other aspects of social support. Neglecting the multitudes of support sources used by ambulance personnel increases the risk of overlooking important dynamics in support utilizations and thus a factor that could be important for the interpretation of the effects of specific support interventions or support measures utilized in preventive strategies. Our results contrast past findings of social support and PTSS by finding a clear exposure response pattern between use of support utilization and PTSS. We do not necessarily believe this to be an indicator of negative consequences of using social support, although this can be a possible contributor to the effects. As prior studies have found contrasting or no effects of social support on PTSS by measuring perceived social support (41, 42, 44), distinguishing perception or satisfaction and utilization of support and it’s patterns over time might contribute with another nuance to the evidence on the area.

In all multiple regression analyses, frequency of traumatic incidents at work was statistical significantly associated with PTSS with moderate effect sizes.

The results corroborate support extant research of repeated exposure to critical incidents as a risk factor of occupational PTSD (12) and of the isolated effects of frequency of exposure for ambulance personnel specifically (14). As the content of exposure in ambulance work is often stressful (73), our results support that frequency of exposure might serve as an isolated measure for risk assessing work-related PTSS apart from the character of the event itself. Furthermore, our results support the findings of Wild and Chang (74) that higher exposure to critical incidents at work are stronger associated with symptom severity than prior civil trauma, as civil trauma did not have substantial effect on PTSS to be included as a confounder in our multiple regression analyses (75). Intensity of this work year’s most traumatizing incident and actively trying not to think of this year’s trauma at work both had statistically significant, positive associations with PTSS in all analyses.

### Social support utilization moderates frequency of exposure to critical events and PTSS

For our second hypothesis, we found that utilized support served as a moderator of the association between frequency of exposure to critical events at work and PTSS. The effect was positive, indicating that social support use can amplify the effect of frequency of exposure to critical incidents at work on PTSS for this group. Reti et al. (45) studied if received or perceived social support moderated the effect of traumatic exposure on PTSS (47). They found no interaction effect on the association by either measure. Their measure of received support was constructed by a summary of a variety of supportive actions received from unknown support source, thus differing from the character of our measure. Also, their exposure variable was direct trauma and not frequency of exposure, which might also explain the contrasting findings. Our results could thus indicate that to understand the effect of frequency of exposure to critical events in ambulance work on PTSS, it is relevant to consider the individual’s use of social support.

Our results could be interpreted as reflecting social support’s potentially detrimental effect for the mental health of ambulance personnel. We do, however, suggest other possible, underlying mechanisms of the positive moderation effect.

Our result could reflect that employees accessing and using more formal support have higher levels of PTSS, possibly due to continuous exposure and lacking resources to seek effective support in time. This may indicate that pressing needs to improve one’s mental health may motivate the rescuer in need and his/her peers and organization to prioritize help. The results might also indicate that more severely impacted professionals seek more support. Hence, the use of support when more distressed could indicate a well-functioning support system, particularly under conditions such as this, where there is limited problems with PTSD. The stratified analyses showed that seeking more formal sources of support and not informal support increases likelihood to report higher levels of PTSS, when adjusting for confounders. Thus, the main associations between support utilization and PTSS might be driven by the use of formal support. This seems plausible, as incentive to seek formal support can be expected, when one is more distraught, thus reversing the causality of our hypothesis.

The results regarding use of formal support could be an expression of a stepped care principle, where employees are gradually introduced to more formal support types, as the level of symptom increases. This interpretation is also supported by the fact that in order to elicit crisis support and formal peer support, the employee must usually go via manager or a general practitioner, why accumulation of several utilized formal support types is dependent on earlier utilization of some formal support types.

On the other hand, our results might also be based on a mechanism where the employees in the ambulance profession have an insufficient or untimely engagement in the available support, and that formal support is sought when PTSS symptoms are more persistent or severe. This would support past findings implying a general reluctance towards seeking help due to e.g. fear of stigma (5, 34) and fear of overwhelming peers (33) or not finding time to seek support or not knowing where to get proper help (35). Other studies have shown, that job strain of both the individual, colleagues and managers can lead to prioritizing accessible *ad hoc* support over formal support initiatives which are more often planned and scheduled, which could explain why formal support are only sought, when symptom levels are high enough to hamper the daily functioning at work (3, 34, 37–39, 76). Ambulance services in Denmark have generally been short-staffed recent years (48). The same tendencies are also reported in other countries e.g. the UK (77) and the US (78). This could indicate a risk of increased job strain possibly interfering with ambulance workers’ possibility of seeking support at work. However, these possible and purely hypothetical causal mechanisms need to be corroborated in future research.

### Informal support types are more used than formal support types

As hypothesized in our third hypothesis, we found a clear pattern of informal support being used more than formal support. This is consistent with past qualitative research, where informal support, e.g. talking with the work partner on the way home in the truck, was favored by ambulance rescuers, partially due to feeling more private (25, 40).

Use of informal support from managers was prevalent in our sample, as 35% of the respondents used this type of support. This is in contrast to previous, qualitative findings that managerial support was not preferred and could be considered a source of stress instead (38). As our data only provides information on whether a support type has been used or not, we cannot assess possible negative attitudes towards managerial support. The fact that more than a third of the respondents seek informal leader support indicates that this type of managerial support might be preferred over more formal support types.

Where informal support was overall used by most of our respondents, the pattern was reversed for formal support. Here, 188 (49.1%) of the respondents had not utilized any form of formal support during the past year. This could reflect that half of the respondents do not feel the need of further support. Resiliency and experience has been promoted as a characteristic and potential protective factor of first responders (2, 17, 75) that might affect incentive to seeking further support, e.g. due to self-efficacy (75).

Other studies argue that refraining from using formal support is the result of an inherent working culture that fosters a worry of being judged or sanctioned by the workplace or colleagues, or that more specialized support does not appear attainable to the employees (25, 26, 33). Thus, the results overall supported prior qualitative findings.

## Limitations

Our study has limitations. Due to the cross-sectional design, causal inferences cannot be drawn from the results. Prospective studies may disclose whether higher level of exposure and PTSS leads to increased utilized support over time or whether the utilized support has a negative effect on PTSS. The possibility of periods of high exposure and social support utilization may vary sequentially multiple times over a year, why longitudinal studies with repeated measures are needed in order to clearly establish the time dependent dynamics of exposure, support and PTSS. However, as the study is the first to investigate social support as number of utilized support types, we chose a cross-sectional design to evaluate the relevance of this way of measuring social support.

A possible bias for this type of study is selection bias. An ambulance study with a similar response rate have proposed that the possibility of selection bias could affect the estimation of levels of health symptoms due to measuring a working population (44). This could potentially compromise the generalizability of our results.

The response rate and sample characteristics are comparable to previous quantitative studies on ambulance personnel’s mental health (21, 44, 79). Our sample represents 54.5% of the organization, close to 12% of the entire ambulance force in Denmark, and was overall comparable with the organization in general. The representativeness of the sample increases the generalizability and practical relevance of the study. We therefore believe that the study’s results are not a direct product of selection bias.

It is worth noting that the mean level of PTSS was low with the majority score no or very few symptoms, and few scoring higher levels of PTSS. Our results thus reflect somewhat minor differences in symptom levels.

Common method bias is a risk due to the use of self-reported data only. Transient mood-state, social desirability and consistency motif could affect the responses (80). The survey method was chosen to capture phenomena of mostly intrapsychic character (81). Thus a more subjective measure like survey is suitable for capturing these sort of phenomena. Further, common method bias should not affect the interaction effects in our results (82). It is also unclear whether common method bias favorizes the null hypothesis or not (83). Therefore the results are not necessarily inflated in relation to the zero hypothesis. Inclusion of both objective data and survey data could decrease risk of bias in future studies.

Recall bias may also pose a limitation to our data on exposure as the respondents were asked to assess frequency of critical incidents at work during the past year and their use of social support. This might cause uncertainty of the rating due to recall bias in cases where certain types of exposure are highly frequent and considered a common task, and recollection of social support episodes. However, previous research has explored different relationships between the demands, control, and support dimensions at work and indicators of mental health with 1-year, 2-year, 3-year, and 4-year time lags. The results revealed that the strongest effects were found for a 1-year time lag (84). This risk of potential bias was managed by asking about specific categories of exposure and support sources, other than the most significant to the respondent and by using validated questionnaires. Future studies could profit from measuring over shorter periods, thus inquiring about episodes within a shorter time frame, in order to reduce recall bias.

## Future directions for research

Attention to lower levels of PTSS can be relevant when studying working ambulance personnel with reference to findings that habituation of PTSS among ambulance personnel can lead to a higher risk of PTSD over time (21). Prospective data of the occupational group could disclose potential differences and progression in symptom levels over time.

We believe our results can serve as a foundation for further preventive initiatives and research in this field by providing a quantitative overview of utilized social support as a predictor for PTSS. Our results indicate a need for further studies on the patterns of help seeking over time and its’ effects.

Also, other factors can be relevant for the association between social support utilization and PTSS. Future studies could include personality factors related to help seeking behavior such as personality traits, locus of control, and resiliency to ascertain how support and PTSS are interconnected. The effects of frequency of certain types of exposure, such as emotionally demanding situations, situations involving children, or fatalities on social support utilization, could also serve as a relevant future research question.

Further research with prospective data could explore how the specific types and patterns of support sources utilized affects the development of posttraumatic stress symptoms. Finally, the modifying effect of intensity of the critical event might be interesting to investigate, since motivation for seeking support and granting of support such as debriefing and formal crisis support are often given due to the intensity of the event. Effectiveness and satisfaction of the social support could be factors contributing to the association between social support utilization and PTSS. Appraisal and character of the event, the way the traumatic exposure was managed, and coping self-efficacy may also be underlying mechanisms, affecting utilization of support. Further investigation of these factors would be relevant to clarify the relation between social support usage and PTSS among ambulance personnel.

## Conclusion

This study found that utilization of multiple support types was associated with higher level of PTSS, though the mean level of PTSS of the sample was low. Number of utilized social support types had a statistically significant moderating effect on the association between frequency of exposure to critical incidents and PTSS, albeit also in a positive direction. Stratified analyses revealed that the effects of support utilization were likely driven by the effects of formal support utilization.

This study’s contribution to the field of studies on first responders’ mental health is the measurement of utilized support types and its association with PTSS and trauma exposure. Social support among ambulance personnel’ needs to be investigated with attention to the multitude of concurrent and different patterns of social support use that constitutes professional life. We suggest that to fully understand the effects of social support in the context of continuous work exposure to trauma, usage of multiple sources of social support and effects over time must be taken into account.

## Data availability statement

The datasets presented in this article are not readily available because the dataset contains sensitive clinical and personal information that might identify individual participants. We do not have the ethics committee’s or our participants’ consent to grant access to the collected data to third parties outside the research project. Requests to access the datasets should be directed to pernille.melander@rsyd.dk.

## Ethics statement

Written informed consent was obtained from the individual(s) for the publication of any potentially identifiable images or data included in this article.

## Author contributions

PM: Conceptualization, Data curation, Formal analysis, Investigation, Methodology, Software, Visualization, Writing – original draft, Writing – review & editing. MV: Writing – review & editing. NL: Writing – review & editing. LA: Writing – review & editing. AE: Writing – review & editing. JP-T: Conceptualization, Funding acquisition, Methodology, Project administration, Resources, Supervision, Validation, Writing – review & editing.
